# Interactive effect of 24-epibrassinolide and silicon alleviates cadmium stress via the modulation of antioxidant defense and glyoxalase systems and macronutrient content in *Pisum sativum* L. seedlings

**DOI:** 10.1186/s12870-018-1359-5

**Published:** 2018-07-16

**Authors:** Sumira Jan, Mohammed Nasser Alyemeni, Leonard Wijaya, Pravej Alam, Kadambot H. Siddique, Parvaiz Ahmad

**Affiliations:** 1 ICAR- Central Institute of Temperate Horticulture, Rangreth, Air Field, Srinagar, Jammu, Kashmir India; 2 Department of Botany and Microbiology, Faculty of Science, King Saud University, Riyadh, 11451 Saudi Arabia; 3 Biology Department, College of Science and Humanities, Prince Sattam bin Abdulaziz University, Alkharj, Kingdom of Saudi Arabia; 4 The UWA Institute of Agriculture and School of Agriculture & Environment, The University of Western Australia, LB 5005, Perth, WA 6001 Australia; 5 Department of Botany, S.P. College, Srinagar, Jammu, Kashmir 190001 India

**Keywords:** *Pisum sativum*, Cadmium stress, 24-Epibrassinolide, Silicon, Lipid peroxidation, Antioxidants, Glyoxalase system

## Abstract

**Background:**

This study assessed the effects of 24-epibrassinolide (EBL, 10^–7^M) and silicon (2 mM) on the alleviation of cadmium (Cd, 150 mg L^–1^) toxicity in *Pisum sativum* L. seedlings via the modulation of growth, antioxidant defense, glyoxalase system, and nutrient uptake.

**Results:**

Shoot and root lengths declined by 46.43% and 52.78%, respectively, following Cd stress. Shoot and root dry weights also declined with Cd toxicity. Biochemical and physiological aspects exhibit significant decline including total chlorophyll (33.09%), carotenoid (51.51%), photosynthetic efficiency (32.60%), photochemical quenching (19.04%), leaf relative water content (40.18%), and gas exchange parameters (80.65%). However, EBL or Si supplementation alone or in combination modulates the previously mentioned parameters. Cadmium stress increased proline and glycine betaine (GB) contents by 4.37 and 2.41-fold, respectively. Exposure of plants to Cd stress increased the accumulation of H_2_O_2_, malondialdehyde content, electrolyte leakage, and methylglyoxal, which declined significantly with EBL and Si supplementation, both individually and in combination. Similarly, Cd stress adversely affected enzymatic and non-enzymatic antioxidants, but EBL and/or Si supplementation maintained antioxidant levels. Glyoxalase I (GlyI) accumulated after Cd stress and increased further with the application of EBL and Si. However, GlyII content declined after Cd stress but increased with supplementation of EBL and Si. Cadmium accumulation occurred in the following order: roots > shoots>leaves. Supplementation with EBL and Si, individually and in combination reduced Cd accumulation and enhanced the uptake of macronutrients and micronutrients in shoots and roots, which declined with Cd toxicity.

**Conclusion:**

The application of 24-EBL and Si, individually and in combination, alleviated the adverse effects of Cd by improving growth, biochemical parameters, nutrient uptake, osmolyte accumulation, and the anti-oxidative defense and glyoxalase systems in *Pisum sativum* seedlings.

## Background

Heavy metal contamination is a serious threat caused by anthropogenic activities such as mining, wastewater and sewage sludge utilization for irrigationpurposes, phosphate fertilizer application, and increased vehicular and industrial emissions [1–3]. Heavy metal contamination causes morphological, physiological, biochemical, and ultra-structural alterations in plants [4]. Cadmium (Cd) is among the comparatively mobile heavy metals in soil and is highly toxic to both plants and animals [5, 6]. Cadmium accumulates progressively in humans via the food chain [6–9], leading to human disorders such as Itai-Itai disease, cancer, neurotoxicity, and nephrotoxicity [5]. Vegetables and cereals are the primary food sources for the world’s population. Accumulation of Cd in vegetables is noted when fields are irrigated with wastewater and sewage sludge in peri-urban areas [10]. Cd toxicity reduces plant growth, biomass, photosynthesis, yield, and quality [11, 12]. Further, it impairs mineral nutrition in plants [13, 14]. Several studies have shown that Cd toxicity alters nitrogen metabolism, reduces photosynthetic efficiency caused by impaired chlorophyll synthesis, and reduces carbon fixation [8, 9]. Increased Cd accumulation hampers root morphology, resulting in stunted growth [15], and thus causes oxidative stress in vegetables via the generation of reactive oxygen species (ROS), which damage the antioxidant enzyme system [1, 16]. To tolerate Cd stress, plants have developed advanced tolerance strategies, including osmoprotectant synthesis and antioxidant defense and glyoxalase systems [1, 8, 16–20]. Enzymatic and non-enzymatic antioxidants related to the ascorbate–glutathione cycle have a crucial role in stress tolerance mechanisms in plants [2, 17, 21]. The enzymatic antioxidants include superoxide dismutase (SOD), catalase (CAT), ascorbate peroxidase (APX), glutathione reductase (GR), monodehydroascorbate reductase (MDHAR), dehydroascorbate reductase (DHAR), and glutathione-S-transferase (GST); non-enzymatic antioxidants include ascorbic acid (AsA) and glutathione (GSH). In addition to the antioxidant defense system, the glyoxalase system [glyoxalase I (Gly I) and glyoxalase II (Gly II)] facilitates the detoxification of methylglyoxal (MG) [21, 22]. However, Cd toxicity exhibits diverse specificity within vegetable cultivars and genotypes [23, 24]. Leafy vegetables accumulate more Cd than vegetable roots and tubers [25].

Numerous strategies have been proposed to combat Cd toxicity encompassing the exogenous application of organic and inorganic amendment such as plant growth regulators as well as mineral amendments like silicon and selenium [1, 2, 10, 16–18, 26–28]. Among the phytohormones significance of brassinosteroids (BRs) in alleviation of stress is more expansively reported [2, 29–32]. Mineral amendment in plant media has also been reported of functional significance to alleviate heavy metal toxicity. Silicon (Si) is the one among such minerals that is well documented for the amelioration of various biotic and abiotic stresses [33, 34]. Accumulation of Si under the leaf surface enhances both biotic and abiotic stress tolerance by reducing the transpiration rate and thus restoring water utilization competence [35]. Si bio-fortification is also associated with an improved oxidative defense system and enhanced membrane integrity in plants subjected to various abiotic and biotic stresses [8, 34].

Brassinosteroids are a category of steroidal phytohormones that are present in plant parts, including roots [24]; they modulate an extensive range of physiological responses, including cellular and metabolic functions [8]. Moreover, BRs exhibit diverse ameliorative effects against varied stresses such as thermal stress [36], oxidative damage [37], and heavy metal toxicity [38, 39]. Studies have shown that exogenous BRs alleviated Cd stress in tomato [40], bean [41], tobacco [42], and peanut [43]. The possible relevance of BRs in agriculture is determined primarily by their competence to augment crop yield and modulate stress-induced damage. However, whether Si and 24-epibrassinolide (EBL) can ameliorate Cd-induced physiological and metabolic alterations is still unraveled.

Pea is an important legume crop that is widely cultivated globally for its nutritive value. The indiscriminate use of fertilizers has caused an enormous flow of Cd into pea fields, which has become detrimental to the quality of pea for human and livestock consumption. Numerous health risks are associated with Cd uptake by plants [44]. Keeping in view the alleviation potential of EBL and Si against Cd toxicity, we hypothesize that individual and combined treatment of Si and EBL can modulate biochemical status and anti-oxidant defense system in pea seedlings. Therefore, this study evaluated the effect of EBL and Si, individually and in combination, on the growth, physiology, nutrient uptake, and antioxidant defense and glyoxalase systems in pea plants grown under Cd stress.

## Methods

Seeds of pea (*Pisum sativum* L.) were sterilized using 5% NaOCl for 5 min and washed with double-distilled water. Seeds were pretreated with EBL (10^–7^M) for 8 h. The seeds were sown in pots containing sand, perlite, and peat in a 1:1:1 ratio. After germination, seedlings were thinned to one per pot. From sowing to 10 days of seedling growth, pots were supplemented with 200 mL full-strength Hoagland solution every alternate day [45]. After 10 days, seedlings were treated with Cd (CdSO_4_·8H_2_O; 150 mg L^–1^) in a modified Hoagland solution. Silicon (2 mM, 10mL) was supplemented in the form of Na_2_SiO_3_ with Hoagland solution. Silicon was supplied every alternate day to plants after one week of NaCl treatment up to 40 days. Pots were maintained in a growth chamber set at 26 ± 2 °C/15 ± 2 °C day/night temperatures, 70–75% relative humidity, and an average 18 h light dark photoperiod. After 40 days of treatment, the plants were carefully uprooted and analyzed for different parameters. The biochemical and antioxidant activities were estimated using secondary leaves.

### Determination of growth parameters

Shoot and root lengths were measuredwith a ruler. Shoot and root fresh weights (FW) were determined immediately after harvesting, and dry weights (DW) were determined after oven drying at 70°C for 72 h.

### Determination of pigments

Chlorophyll content in leaves was extracted using dimethyl sulphoxide (DMSO), and absorbance was determined spectrophotometrically at 480, 510, 645, and 663 nm (Beckman 640 D, USA) against DMSO [46]. The total carotenoid content was determined using 80% acetone extracts of the plant material, according to the spectrophotometric method of Lichtenthaler and Wellburn [47].

### Chlorophyll fluorescence

Chlorophyll fluorescence was measured using a portable pulse modulation fluorometer (PAM 2500; Waltz GmbH, Effeltrich, Germany). Leaves from each plant were randomly selected from one replication per treatment and dark-adapted for approximately 10 min (based on the previous experiment),before measuring initial fluorescence (Fo), maximal fluorescence (Fm), actual photochemical efficiency of PSII (Φ PSII), photochemical quenching (qP), and non-photochemical quenching (NPQ) at 1200 μmol m^–2^s^–1^PAR. After the actinic light (AL) source was removedand 3 s of far-red light was applied, the minimal fluorescence of the light-adapted state (Fo′) was obtained. Steady-state fluorescence (Fs) was determined under AL (λ = 665 nm). The relative effective quantum yield of photochemical energy conversion at steady-state photosynthesis was calculated as yield = (Fm′ − Fs)/Fm′, where Fs and Fm′ are the fluorescence at steady-state photosynthesis and maximum fluorescence in the light, respectively. Next, qP, Φ PSII, and NPQ were calculated as (Fm′ − Fs)/(Fm′ − Fo′), (Fm′ − Fs)/Fm′, and (Fm − Fm′)/Fm, respectively [48].

### Determination of H_2_O_2_ content, lipid peroxidation, and electrolyte leakage

Fresh leaf samples (500 mg) were homogenized in 5 mL of trichloroacetic acid (0.1%, w/v), and the homogenate centrifuged at 12,000*g* for 15 min. Next, 0.5 mL supernatant was mixed with 0.5 mL of 10 mM potassium phosphate buffer (pH 7.0) and 1 mL of 1 M potassium iodide. The optical density was recorded at 390 nm [49].

The method of Madhava Rao and Sresty [50] was used to measure lipid peroxidation (formation of malondialdehyde (MDA). Fresh leaf tissue (500 mg) was homogenized in 2.5 mL of trichloroacetic acid (0.1%), followed by centrifugation at 10,000*g*for 10 min. Next, a 1mL aliquot was mixed with 4 mL of 20% trichloroacetic acid containing 0.5% of thiobarbituric acid, and heated at 95°C for 30 min. The mixture was cooledin anice bath and centrifuged again for 15 min at 10,000*g*. The absorbance was measured at 532 nm, and corrections were performedfor unspecific turbidity by subtracting the absorbance at 600 nm.

To estimate electrolyte leakage, 20 leaf discs were immersed in test tubes containing deionized water, and the electrical conductivity was measured (ECa). Subsequently, tubes were heated in a water bath for 25 min at 50°C, and the electrical conductivity (ECb) was measured. The tubes were heated again for 10min at 100°C, and the electrical conductivity (ECc) was measured [51]. Electrolyte leakage (EL) was calculated as follows:

$$ \mathrm{Electrolyte}\ \mathrm{leakage}\ \left(\%\right)=\frac{{\mathrm{EC}}_{\mathrm{b}}-{\mathrm{EC}}_{\mathrm{a}}}{{\mathrm{EC}}_{\mathrm{c}}}\times 100 $$

### Measurement of methylglyoxal level

The method of Wild, et al. [52] was used to estimate methylglyoxal (MG). Fresh leaves (500 mg) were crushed in perchloric acid (5%) and subjected to centrifugation at 4°C for 10 min at 11,000*g*. The collected supernatant was mixed with charcoal to decolorize and then neutralized with saturated potassium carbonate solution. Sodium dihydrogen phosphate and *N*-acetyl-l-cysteine was added to neutralize the supernatant used for MG estimation, and the final volume was madeup to 1 mL. The formation of product *N*-α-acetyl-S-(1-hydroxy-2-oxo-prop-1-yl) cysteine after 10 min was recorded at 288nm using a spectrophotometer (Beckman640D, USA). A known concentration of MG was used to generate the standard curve, and MG was expressed as μmol g^–1^ FW.

### Estimation of physiological parameters

Gas exchange parameters were determined when the plants were 26-weeks-old. Net photosynthesis (*Pn*), CO_2_ assimilation rate (*A*), stomatal conductance (*gs*), transpiration rate (*E*), intercellular CO_2_ concentration (Ci), and CO_2_ resistance (Rs) were determined using an infrared gas analyzer (LCA-4 model; Analytical Development Company, Hoddesdon, England) and the uppermost fully expanded leaves.

### Estimation of leaf relative water content, proline, and glycinebetaine

The relative water content (RWC) in leaves was measured according to Yamasaki and Dillenburg [53], , and calculations were performed using the following formula:

$$ \mathrm{RWC}\ \left(\%\right)=\left(\mathrm{Fresh}\ \mathrm{weight}-\mathrm{Dry}\ \mathrm{weight}/\mathrm{Turgid}\ \mathrm{weight}-\mathrm{Dry}\ \mathrm{weight}\right)\times 100 $$

Proline content was determined following the method of Bates, et al. [54]. Absorbance was recorded spectrophotometrically at 520 nm (Beckman 640 D, USA) using toluene as a blank.

The method of Grieve and Grattan [55] was used to estimate glycine betaine (GB). Absorbance was measured at 365 nm using a spectrophotometer, and calculations were performed using the reference standard for GB (50–200 mg mL^–1^).

### Extraction of enzymes and their assay

Fresh leaves (0.5g) were macerated using a chilled mortar and pestle in phosphate buffer (0.1M, pH 7.5) and ethylenediaminetetraacetic acid (EDTA, 0.5 mM). The homogenate was filtered through four layers of muslin cloth and subjected to centrifugation at 12,000 *g* for 10 min at 4°C. The resulting supernatant was used for the enzyme assays.

Superoxide dismutase (SOD; EC1.15.1.1) activity was determined by measuring the ability of theenzyme to inhibit the photochemical reduction of nitrobluetetrazoliumchloride, as described by Beyer and Fridovich [56]. The absorbance of the reaction mixture was read at 560nm, and one unit of SOD activity (EU) was defined as the amount of enzyme required to inhibit 50% of the NBT photoreduction rate and expressed as EU mg^–1^ protein.

APX (EC 1.11.1.11) was assayed following a reduction in absorbance at 290 nm for 3 min, with the activity expressed as EU mg^–1^ protein [57].

Catalase (CAT; EC 1.11.1.6) activity was measured using the method of Aebi [58], with the change in absorbance recorded at 240 nm for 3 min. CAT activity was expressed as unit mg^–1^ protein.

To estimateglutathione reductase (GR; EC 1.6.4.2) activity, the method of Foyer and Halliwell [59] was used, and the reductionin absorbance measured at 340 nm for 3 min. The GR activity was expressed as EU mg^–1^ protein.

Glutathione-*S*-transferase (GST; EC 2.5.1.18) was estimated following Hasanuzzaman and Fujita [60], and the increase in absorbance measured at 340nm for 3 min witha spectrophotometer (Beckman 640D, USA). The GST activity was expressed as EU mg^–1^ protein.

Monodehydroascorbate reductase (MDHAR; EC 1.6.5.4) activity was estimated following the method of Miyake and Asada [61]. The change in absorbance was observed at 340nm for 3 min, with the activity expressed as EU mg^–1^ protein.

The activity of dehydroascorbate reductase (DHAR; EC 1.8.5.1) was estimated using the method of Nakano, et al. [57]. The absorbance was read at 265nm for 3 min using a spectrophotometer (Beckman640D, USA),with the activity expressed as EU mg^–1^ protein.

Glyoxalase I (EC: 4.4.1.5) activity was estimated according to the method of Hasanuzzaman, et al. [23]. The assay mixture contained 100 mM K-P buffer (pH 7.0), 15 mM magnesium sulfate, 1.7 mM GSH, and 3.5 mM MG. The reaction was started by adding MG; the increase in absorbance was recorded at 240 nm using a spectrophotometer (Beckman 640D, USA) for 1 min, with the activity expressed as μmol min^–1^ mg^–1^ protein.

Glyoxalase II (EC: 3.1.2.6) was estimated using the method of Principato, et al. [62]. The reaction mixture contained 100 mM Tris–HCl buffer (pH 7.2), 0.2 mM DTNB, and 1 mM *S*-d-lactoylglutathione (SLG). The reaction was started by adding SLG, and theabsorbance at 412 nm was measured usinga spectrophotometer (Beckman 640D, USA). The activity was expressed as μmol min^–1^ mg^–1^ protein.

### Non-enzymatic antioxidants

Ascorbate was extracted from fresh leaves (0.8g) in 3mL ice-cold metaphosphoric acid (5%) containing 1mM EDTA and centrifuged at 10,000 rpm for 10 min. The supernatant was distributed in two separate micro centrifuge tubes (400 μl in each) for the assay of total ascorbate (As + DAs) and reduced ascorbate. DAs concentration was then deduced from the difference. To each tube 200 μl of 10% TCA was added and vortexed mixed. 10 μl of NaOH solution was then added to it, mixed and the mixture was centrifuged for 2 min in microcentrifuge. To 200 μl of the supernatant, 200 μl of 150 mM of NaH_2_PO_4_ and 200 μl of water were added. To another 200 μl of supernatant, 200 μl of buffer and 100 μl of 10 μl of 10 mM DDT were added and thoroughly mixed. Then 100 μl of 0.5% N- Ethylmaleimide was added to each tube. Both samples were vortexed mixed and incubated at room temperature for 30 min. To each tube was then added 400 μl of 10% TCA, 400 μl of 44% H_3_ PO_4_, 4μl of 4% bipyridyl and 200 μl of 3% FeCl_3_. After vortexed mixing, samples were incubated at 33°c for 60 min. The supernatant was then used for ascorbate analysis [63], and the absorbance was recorded at 525 nm on uv-vis spectrophotometer (Model Du 640, Beckman, USA).

The method of Yu, et al. [64] was used to estimate the glutathione pool, and standard curves with known concentrations of GSH and GSSG were used for calculations. 0.5 g of fresh leaf was homogenized in 2 ml of 5% sulphosalicylic acid under cold condition. The homogenate was centrifuged at 10,000 rpm for 10 min. 0.5 ml of aliquot was taken in a micro centrifuge tube, to which 0.6 ml of reaction buffer and 40 μl of DTNB was added. Absorbance for determination of GSH was read at 412 nm on uv- vis spectrophotometer (Model DU 640, Beckman, USA) after 2 min. To the same tube 50 μl of NADPH and 2 μl of GR was added for the determination of total glutathione. Subtracting the reduced glutathione from total glutathione determines the oxidized glutathione. The reaction was allowed to run for 30 min. at 25°C. The change in absorbance at 412nm on UV-VIS spectrophotometer (Model DU 640, Beckman, USA) was recorded. Values are corrected for the absorbance of supernatant and DTNB.

### Estimation of Cd and inorganic nutrients

Shoot, root and leaf samples (100 mg each) were digested in H_2_SO_4_/HNO_3_ mixture (1/5, v/v) for 24h and subsequently treated with HNO_3_/HClO_4_mixture (5/1, v/v). Cadmium and other micronutrients (B, Cu, Fe, Mn, Zn) and macronutrients (S, Mg, Ca, K, P) in the solution were determined using a Perkin–Elmer (Analyst Model 300) atomic absorption spectrophotometer. The Cd content was expressed as μmol g^–1^DW and the other nutrients were expressed as μg g^–1^DW.

### Statistical analysis

Data presented arethe means of five replicates with ±SE. Data were analyzed following one-way analysis of variance (ANOVA) using SPSS software version 17. The *P* values at 0.05 were considered significant. Mean values followed by the same lettersdo not significantly differ at *P*< 0.05.

## Results

### Silicon and 24-EBL augmentedplant growth and biomass

**Fig. 1:**
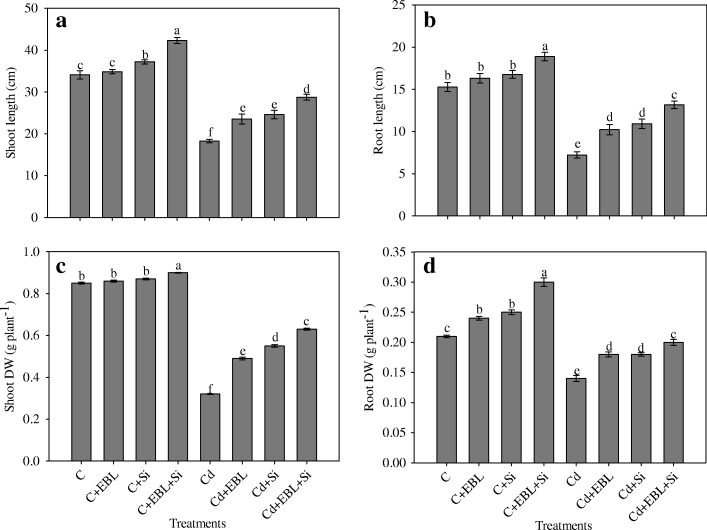
Effect of 24-epibrassinolide and silicon individually and in combination on (**a**) shoot length, (**b**) root length, (**c**) shoot DW and (**d**) root DW in *Pisum sativum* seedlings under Cd stress. Data presented are the means ± SE (*n* = 5). Different letters indicate significant difference at *P* ≤ 0.05

Figure 1a–d shows the growth response (shoot and root DWs and lengths) of pea seedlings to Cd stress (150 mg L^–1^) and the individual and combined effects of EBL and Si. Cadmium stress alone reduced shoot and root lengths by 46.43% and 52.78%, respectively, relative to theuntreated control. In control plants, EBL alone did not affect the shoot and root lengths of control plants. In Cd-stressed seedlings, shoot and root lengths increased by 28.89% and 41.71%, respectively, with EBL, and by 34.70% and 51.31%, respectively, with Si, relative to seedlings exposed to Cd alone (Fig. 1a, b). However, the combined treatment of EBL+Si enhanced shoot and root lengths by 57.47% and 82.66%, respectively, relative to seedlings exposed to Cd alone.

Shoot and root DWs decreased by 52.78% and 62.35%, respectively, in the Cd-alone treatment, relative to the untreated control. However, the Cd + EBL +Si treatment increased shoot and root DWs by 96.87% and 42.85%, respectively, relative to Cd alone, and was more effective than the individual treatments (Fig. 1c, d).

### Silicon and 24-EBL augments pigment content

**Fig. 2:**
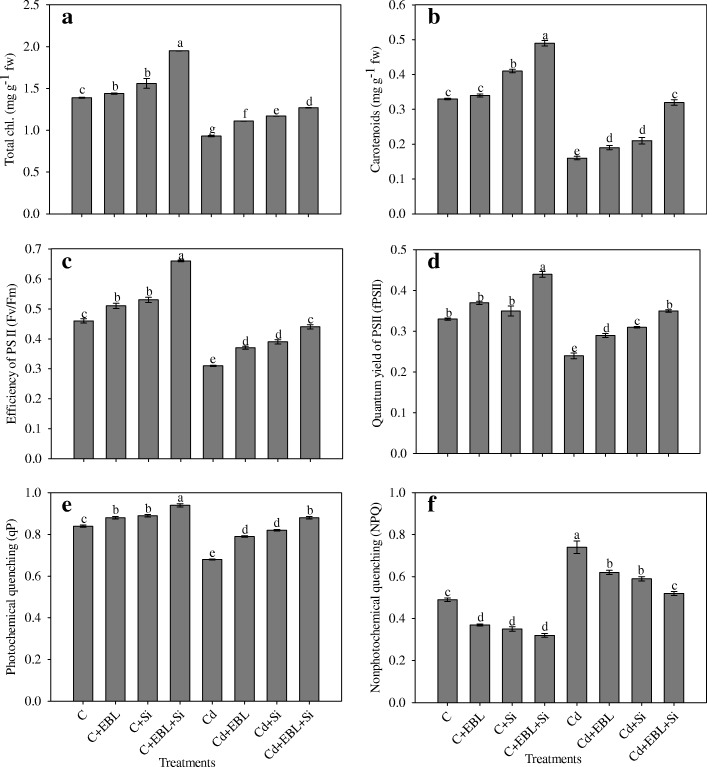
Effect of 24-epibrassinolide and silicon individually and in combination on (**a**) total chlorophyll, (**b**) carotenoid content, (**c**) F_v_/F_m,_ (**d**) ΦPSII, (**e**) qP and (**f**) NPQ in *Pisum sativum* seedlings under Cd stress. Data presented are the means ± SE (*n* = 5). Different letters indicate significant difference at *P* ≤ 0.05

Total chl and carotenoid contents declined by 33.09% and 51.51%, respectively, in Cd-treated plants, relative to the control treatment. In Cd-treated seedlings, total chl and carotenoid contents increased by 19.35% and 18.75%, respectively, with EBL, and by 25.80% and 31.25%, respectively, with Si, relative to seedlings exposed to Cd alone (Fig. 2a, b). The combined Cd + EBL+Si treatment increased total chl and carotenoid contents by 36.55% and 100%, respectively, relative to Cd alone, and was more effective than the individual treatments.

### Silicon and 24-EBL improved photosynthetic efficiency

The effects of Cd, EBL, and Si on chlorophyll fluorescence parameters are shown in Fig. 2c–f. The Cd-alone treatment significantly reduced Fv/Fm (by 32.60%), ΦPSII (by 27.27%), and qP (by 19.04%) but increased NPQ (by 51.02%),relative to untreated controlseedlings. Individual applications of EBL and Si increased Fv/Fm, ΦPSII, and qPanddecreased NPQ in Cd-treated and control seedlings. The combined Cd + EBL+Si treatment was more effective, with Fv/Fm, ΦPSII, and qPincreasing by 41.93%, 45.83%, and 29.41%, respectively, and NPQ decreasing by 29.72%, compared to Cd alone.

### Silicon and 24-EBL modulated physiological status

The Cd-alone treatment reduced all the gas exchange parameters, i.e., *Pn*, *A*, *gs*, and *E* by 46.17%, 56.26%, 80.65%, and 73.00%, respectively, relative to the control seedlings (Table 1). The Cd +EBL treatment increased *Pn* by 24.61%, *A* by 27.67%, *gs* by 137.28%, and *E* by 93.18%, relative to Cd alone. The Cd + Si treatment also enhanced all the parameters. The combined Cd + EBL+Si treatment had a more pronounced effect, increasing *Pn* by 64.67%, *A* by 68.53%, *gs* by 425.42%, and *E* by 165.90%, relative to Cd alone.

**Table 1:** Effect of 24-epibrassinolide and silicon individually and in combination on gas exchange parameters (*Pn*, *A, gs*, *E*) (E) RWC, Proline and glycine betaine content in *Pisum sativum* seedlings under Cd stress

Treatment	Net photosynthesis rate *Pn* (μM m^-1^ S^-1^)	CO_2_ assimilation rate *A* (μM CO_2_ m^-2^ S^-1^)	Stomatal conductance *gs* (mM CO_2_ m^-2^ S^-1^)	Transpiration rate E (mM H_2_O m^-2^ S^-1^)	Proline (μg g^-1^ fw)	GB (μg g^-1^ fw)	RWC (%)	
C	12.15±0.075^c^	13.88±0.031^d^	305±1.19^d^	1.63±0.014^c^	26.31±0.23^f^	2.04±0.011^e^	92.77±0.3^b^	
C+EBL	12.37±0.073^c^	14.03±0.027^c^	380±2.671^b^	1.77±0.022^b^	27.44±0.13^e^	2.05±0.016^e^	93.52±0.508^b^	
C+Si	13.01±0.079^b^	14.95±0.08^b^	385±1.682^b^	1.75±0.018^b^	27.15±0.15^e^	2.17±0.03^d^	93.77±0.316^b^	
C+EBL+Si	14.21±0.091^a^	16.07±0.092^a^	401±2.753^a^	1.88±0.029^a^	28.37±0.25^d^	2.23±0.039^d^	95.42±0.125^a^	
Cd	6.54±0.022^f^	6.07±0.025^g^	59±3.695^g^	0.44±0.028^f^	115±1.7^c^	4.93±0.036^c^	55.49±0.511^e^	
Cd+EBL	8.15±0.034^e^	7.75±0.044^f^	140±3.391^e^	0.85±0.011^e^	125±1.59^b^	5.74±0.05^b^	74.21±0.276^d^	
Cd+Si	8.65±0.035^e^	7.81±0.043^f^	120±2.372^f^	0.89±0.012^e^	129±1.62^b^	5.87±0.053^b^	76.84±0.588^d^	
Cd+EBL+Si	10.77±0.055^d^	10.23±0.063^e^	310±1.12^c^	1.17±0.018^d^	154±1.74^a^	6.14±0.072^a^	88.42±0.155^c^	

Data presented are the means ± SE (*n* = 5). Different letters next to the number indicate significant difference at *P* ≤ 0.05

### Silicon and 24-EBL ameliorated proline, glycine betaine and leaf relative water contents

Cadmium stress alone reduced RWC by 40.18%, relative to the control. The Cd + EBL, Cd + Si, and Cd + EBL + Si treatments increased RWC by 33.73%, 38.47%, and 59.34%, respectively, relative to Cd alone (Table 1).

The Cd-alone treatment increased proline and GB contents by 4.37-fold and 2.41-fold, respectively, relative to the control. These values were further increased with EBL (1.08-fold for proline and 1.16-fold for GB), Si (1.12-fold and 1.19-fold, respectively) and EBL + Si combined (1.33-fold and 1.24-fold, respectively) (Table 1).

### Silicon and 24-EBL reduced hydrogen peroxide MDA contents, and electrolyte leakage and methyl glyoxalase

**Fig. 3:**
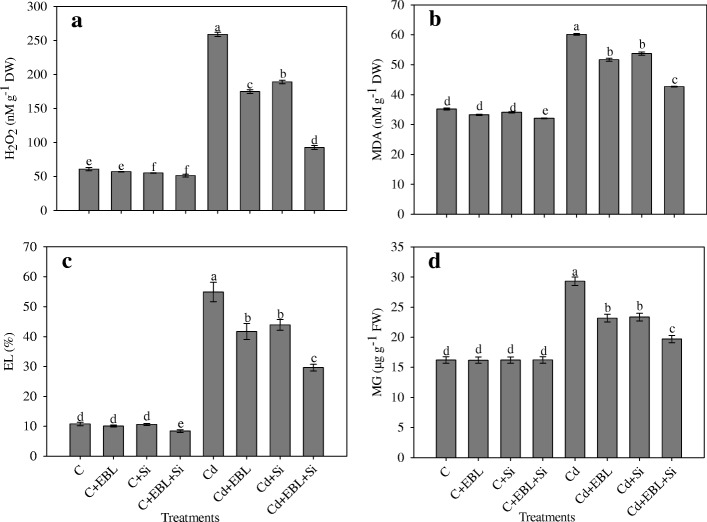
Effect of 24-epibrassinolide and silicon individually and in combination on (**a**) H_2_O_2_ content, (**b**) MDA content (**c**) EL and (**d**) MG in *Pisum sativum* seedlings under Cd stress. Data presented are the means ± SE (*n* = 5). Different letters indicate significant difference at *P* ≤ 0.05

Cadmium stress alone increased H_2_O_2_ production by 325.49%, relative to the control seedlings. The Cd + EBL, Cd + Si, and Cd + EBL + Si treatments decreased H_2_O_2_ by 27.02%, 32.43%, and 64.24%, respectively, relative to Cd alone (Fig. 3a).

Cadmium stress alone increased lipid peroxidation (estimatedfrom MDA content) by 70.71%, relative to the control seedlings. The Cd + EBL, Cd + Si, and Cd + EBL + Si treatments decreased MDA content by 10.63%, 14.00%, and 29.03%, respectively, relative to Cd alone (Fig. 3b).

Cadmium stress alone increased electrolyte leakage by 409.93%, relative to the control. The Cd + EBL, Cd + Si, and Cd + EBL + Si treatments reduced electrolyte leakage by 20.04%, 24.03%, and 46.06%, respectively, relative to Cd alone (Fig. 3c).

Cadmium stress alone increased MG accumulation by 80.70%, relative to the control. The Cd + EBL, Cd + Si, and Cd + EBL + Si treatments decreased MG by 20.94%, 20.33%, and 32.75%, respectively, relative to Cd alone (Fig. 3d).

### Silicon and 24-EBL modulated antioxidant activity

**Fig. 4:**
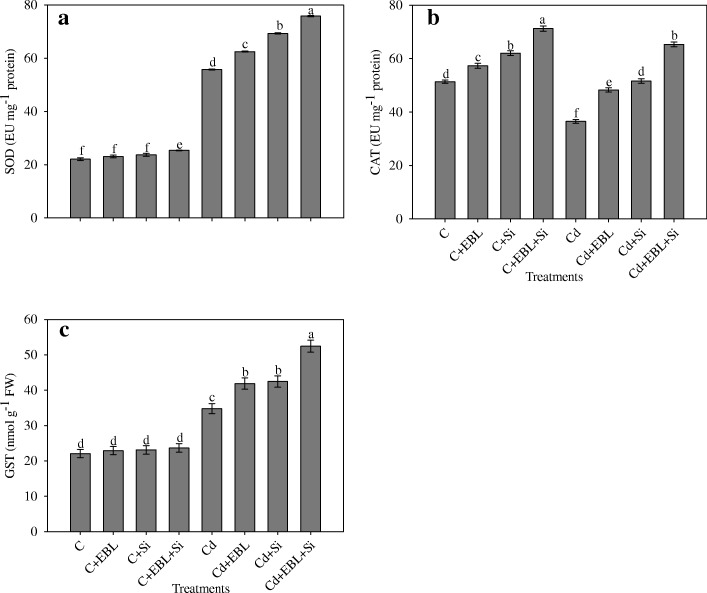
Effect of 24-epibrassinolide and silicon individually and in combination on (**a**) SOD, (**b**) CAT and (**c**) GST in *Pisum sativum* seedlings under Cd stress. Data presented are the means ± SE (*n* = 5). Different letters indicate significant difference at *P* ≤ 0.05

The Cd-alone treatment increased SOD activity by 152.92%, relative to the control treatment. The Cd + EBL, Cd + Si, and Cd + EBL + Si treatments further enhanced this activity by 12.00%, 24.23%, and 35.93%, respectively (Fig. 4a).

Cadmium stress alone increased CAT activity by 28.96%, relative to the control treatment. The Cd + EBL, Cd + Si, and Cd + EBL + Si treatments further enhanced this activity by 32.31%, 41.45%,and78.91%, respectively (Fig. 4b).

Cadmium stress alone increased GST activity by 57.51%, relative to the control. The Cd + EBL, Cd + Si, and Cd + EBL + Si treatments further enhanced this activity by 20.44%, 22.11%, and 50.80%, respectively (Fig. 4c).

**Fig. 5:**
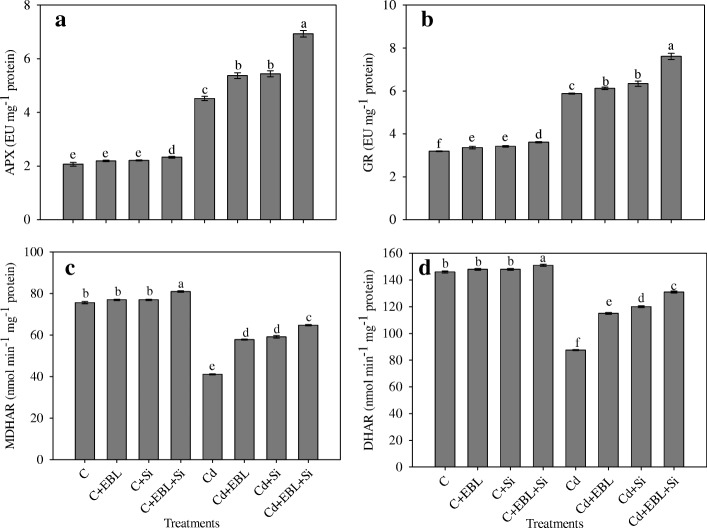
Effect of 24-epibrassinolide and silicon individually and in combination on (**a**) APX, (**b**) GR, (**c**) MDHARand (**d**) DHAR in *Pisum sativum* seedlings under Cd stress. Data presented are the means ± SE (*n* = 5). Different letters indicate significant difference at *P* ≤ 0.05

The Cd-alone treatment increased APX activity by 118.35%, relative to the control. The Cd + EBL, Cd + Si, and Cd + EBL + Si treatments further enhanced this activity by 18.80%, 20.35%, and 53.31%, respectively (Fig. 5a).

Cadmium stress alone increased GR activity by 84.32%, relative to the control. The Cd + EBL, Cd + Si, and Cd + EBL + Si treatments further enhanced this activity by 4.08%, 7.82%, and 29.42%, respectively (Fig. 5b).

The Cd-alone treatment reduced MDHAR and DHAR activities (Fig. 5c, d) by 45.63% and 40.03%, respectively, relative to the control. The Cd + EBL treatment increased the activity of MDHAR by 40.61% and DHAR by 31.35%, relative to the Cd-alone treatment. The combined Cd+EBL+Si treatment further enhanced MDHAR and DHAR activities by 57.56% and 49.62%, respectively, relative to the Cd-alone treatment.

**Fig. 6:**
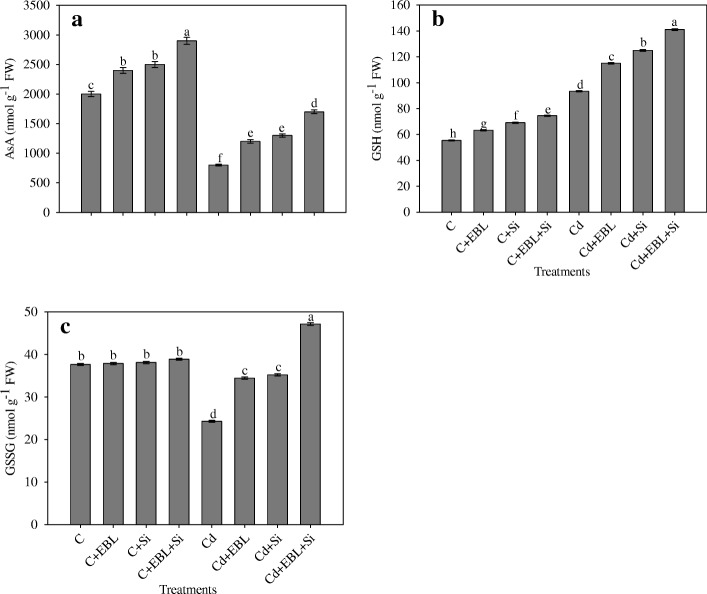
Effect of 24-epibrassinolide and silicon individually and in combination on (**a**) AsA, (**b**) GSH and (**c**) GSSG in *Pisum sativum* seedlings under Cd stress. Data presented are the means ± SE (*n* = 5). Different letters indicate significant difference at *P* ≤ 0.05

Cadmium stress alone reduced the AsA content by 60.00%, relative to the control (Fig. 6a). The Cd + EBL, Cd + Si, and Cd + EBL + Si treatments improved AsA accumulation by 50.00%, 62.50%, and 112.50%, respectively, relative to the Cd-alone treatment.

The Cd-alone treatment increased GSH content by 68.61%, relative tothe control. The Cd + EBL, Cd + Si, and Cd + EBL + Si treatments further enhanced GSH content by 23.08%, 33.79%, and 50.91%, respectively, relative to the Cd-alone treatment (Fig. 6b).

Cadmium stress alone reduced GSSG content by 35.43%,relative to the controls. The Cd + EBL, Cd + Si, and Cd + EBL + Si treatments further enhanced GSSH content by 41.71%, 44.83%, and 56.97%, respectively, relative to the Cd-alone treatment (Fig. 6c).

### Silicon and 24-EBL maintained Gly I and Gly II activities

**Fig. 7:**
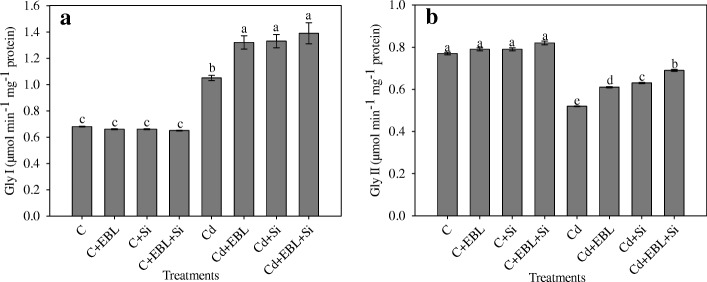
Effect of 24-epibrassinolide and silicon individually and in combination on (**a**) Gly I and (**b**) Gly II in *Pisum sativum* seedlings under Cd stress. Data presented are the means ± SE (*n* = 5). Different letters indicate significant difference at *P* ≤ 0.05

Cadmium stress alone enhanced Gly I activity by 54.41%, relative to the control. The Cd + EBL, Cd + Si, and Cd + EBL + Si treatments further increased this activity by 25.71%, 26.66%, and 32.38%, respectively, relative to Cd alone (Fig. 7a).

TheCd-alone treatment reduced Gly II activity by 32.46%, relative to the control. The Cd + EBL, Cd + Si, and Cd + EBL + Si treatments enhanced this activity by 17.30%, 21.15%, and 32.69%, respectively, relative to Cd alone (Fig. 7b).

### Silicon and 24-EBL reduced Cd accumulation

Cadmium accumulated in different plant parts in the following order: roots > shoots> leaves. EBL supplementation to Cd-stressed seedlings decreased Cd accumulation in the roots, shoots, and leaves by 38.69%, 28.47%, and 48.56%, and similar values were observed after Si supplementation. The combined Cd+EBL+Si treatment further reduced Cd accumulation by 60.15% in roots, 48.63% in shoots, and 68.42% in leaves, relative to Cd alone (Table 2).

**Table 2:** Effect of 24-epibrassinolide and silicon individually and in combination on accumulation of Cd in root, shoot and leaf in *Pisum sativum* seedlings under Cd stress

Treatments	Root Cd (μg g^-1^ FW)	Shoot Cd (μg g^-1^ FW)	Leaf Cd (μg g^-1^ FW)
C	ND	ND	ND
C+EBL	ND	ND	ND
C+Si	ND	ND	ND
C+EBL+Si	ND	ND	ND
Cd	29.64±0.28^a^	10.22±0.13^a^	4.18±0.03^a^
Cd+EBL	18.17±0.20^b^	7.31±0.07^b^	2.15±0.005^b^
Cd+Si	17.55±0.18^b^	7.37±0.07^b^	2.08±0.005^b^
Cd+EBL+Si	11.81±0.12^c^	5.25±0.04^c^	1.32±0.001^c^

Data presented are the means ± SE (*n* = 5). Different letters next to the number indicate significant difference at *P* ≤ 0.05

### Silicon and 24-EBL enhanced mineral uptake

The Cd-alone treatment impaired mineral uptake in the shoots and roots (Table 3). In the shoots, cadmium reduced S, Mg, Ca, P, and K contents by 34.69%, 58.33%, 43.47%, 48.62%, and 57.55%, respectively, relative to the control. Supplementation with EBL or Si to Cd-treated plants resulted in smallerreductions in the above macronutrients; however, the combined Cd + EBL+Si treatment enhanced S uptake by 41.76%, Mg by 114.28%, Ca by 56.92%, K by 47.88%, and P by 64.47%, relative to their respective levels with Cd alone. In the roots, the Cd-alone treatment reduced S, Mg, Ca, K, and P contents by 48.36%, 40.85%, 42.78%, 51.64%, and 51.00%, respectively, relative to the control. Supplementation with EBL or Si to Cd-treated plants enhanced the uptake of these elements, but the combined Cd+EBL+Si treatment was more effective, enhancing S uptake by 56.08%, Mg by 37.47%, Ca by 67.82%, K by 94.97%, and P by 56.12%, relative to Cdalone (Table 3).

**Table 3:** Effect of 24-epibrassinolide and silicon individually and in combination on macronutrients (S, Mg, Ca, K and P) in shoot and root in *Pisum sativum* seedlings under Cd stress

Treatments(μg g^-1^ DW)	Shoot S	Shoot Mg	Shoot Ca	Shoot K	Shoot P	Root S	Root Mg	Root Ca	Root K	Root P
C	95.26±0.905^c^	252±0.95^c^	115±0.45^c^	691±2.885^c^	450±3.72^d^	366±0.942^d^	776±1.26^d^	201±0.762^c^	1152±2.43^d^	200±0.945^c^
C+EBL	99.05±0.985^b^	262±0.976^b^	122±0.458^b^	705±2.96^b^	498±3.91^c^	385±0.987^b^	791±0.302^b^	211±0.77^b^	1240±2.59^b^	225±0.995^b^
C+Si	99.56±0.995^b^	259±0.966^b^	120±0.654^b^	702±1.95^b^	508±4.06^b^	376±0.955^c^	788±0.586^c^	200±0.752^c^	1215±2.51^c^	224±0.992^b^
C+EBL+Si	115±1.135^a^	267±0.99^a^	129±0.47^a^	725±3.085^a^	525±4.32^a^	396±1.0675^a^	820±1.422^a^	225±0.788^a^	1320±2.63^a^	231±1.037^a^
Cd	62.21±0.69^g^	105±0.422^g^	65±0.308^g^	355±1.445^g^	259±2.25^h^	189±0.5975^h^	459±0.73^h^	115±0.438^g^	557±1.262^h^	98±0.502^f^
Cd+EBL	76.49±0.575^e^	189±0.662^e^	89±0.376^e^	466±1.905^e^	347±3.37^g^	266±0.7375^f^	587±1.146^f^	182±0.59^e^	889±1.792^f^	138±0.592^e^
Cd+Si	73.54±0.545^f^	173±0.636^f^	83±0.35^f^	452±1.88^f^	369±3.48^f^	259±0.7175^g^	523±1.042^g^	174±0.542^f^	823±1.754^g^	139±0.612^e^
Cd+EBL+Si	88.19±0.86^d^	225±0.844^d^	102±0.412^d^	525±2.11^d^	426±3.27^e^	295±0.78^e^	631±1.216^e^	193±0.62^d^	1086±2.274^e^	153±0.657^d^

Data presented are the means ± SE (*n* = 5). Different letters next to the number indicate significant difference at *P* ≤ 0.05

The Cd-alone treatment reduced the uptake of micronutrients in the shoots and roots (Table 4). In the shoots, Cd alone reduced B, Cu, Fe, Mn, and Zn by 45.00%, 28.48%, 27.05%, 56.07%, and 37.58%, respectively, relative to thecontrol. Supplementation with EBL or Si, individually or in combination, enhanced the uptake of these micronutrients into shoots. In the roots, Cd alone reduced B, Cu, Fe, Mn, and Zn by 43.64%, 31.39%, 34.52%, 40.07%, and 41.32%, respectively, relative to the control. The Cd + EBL+Si treatment was more effectiveat enhancing micronutrient uptake than the individual treatments, with increases of 64.54% in B, 35.68% in Cu, 36.88% in Fe, 39.37% in Mn, and 53.28% in Zn, relative to Cd alone.

**Table 4:** Effect of 24-epibrassinolide and silicon individually and in combination on micronutrients (B, Cu, Fe, Mn and Zn) in shoot and root in *Pisum sativum* seedlings under Cd stress

Treatments (μg g^-1^ DW)	Shoot B	Shoot Cu	Shoot Fe	Shoot Mn	Shoot Zn	Root B	Root Cu	Root Fe	Root Mn	Root Zn
C	40.31±0.312^b^	31.35±0.582^b^	170±0.622^b^	30.21±0.695^c^	39.27±0.298^b^	28.78±0.262^b^	68.67±0.514^b^	530±3.34^b^	98.7±0.95^c^	46.15±0.426^d^
C+EBL	41.11±0.316^b^	32.27±0.286^b^	175±0.632^b^	37.25±0.75^b^	40.52±0.304^a^	30.21±0.276^a^	72.36±0.52^a^	540±5.34^b^	105±0.98^b^	48.22±0.442^c^
C+Si	41.52±0.322^b^	33.07±0.288^b^	175±0.632^b^	35.11±0.74^b^	41.12±0.306^a^	30.88±0.282^a^	72.91±0.524^a^	546±1.35^b^	104±0.98^b^	50.81±0.464^b^
C+EBL+Si	43.46±0.332^a^	35.71±0.302^a^	194±0.662	42.39±0.805^a^	43.07±0.312^a^	30.95±0.284^a^	74.25±0.534^a^	555±1.37^a^	115±1.02^a^	53.15±0.494^a^
Cd	22.17±0.232^e^	22.42±0.204^e^	124±0.438^e^	13.27±0.49^f^	24.51±0.244^e^	16.22±0.17^e^	47.11±0.424^e^	347±1.03^e^	59.15±0.79^f^	27.08±0.244^g^
Cd+EBL	26.53±0.942^d^	25.77±0.256^d^	152±0.576^d^	24.29±0.62^e^	29.55±0.656^d^	19.35±0.202^d^	55.32±0.466^d^	398±3.14^d^	78.62±2.85^e^	36.91±0.35^f^
Cd+Si	29.11±0.448^d^	25.91±0.26^d^	153±0.574^d^	22.77±0.595^e^	31.47±0.468^d^	20.16±0.222^d^	57.08±0.47^d^	403±3.12^d^	71.25±1.83^e^	37.61±0.354^f^
Cd+EBL+Si	33.42±0.278^c^	28.51±0.27^c^	165±0.596^c^	27.03±0.665^d^	35.26±0.282^c^	26.69±0.24^c^	63.92±0.484^c^	475±2.2^c^	82.44±0.88^d^	41.51±0.39^e^

Data presented are the means ± SE (*n* = 5). Different letters next to the number indicate significant difference at *P* ≤ 0.05

## Discussion

### Silicon and 24-EBL augmented plant growth and biomass

In this study, the effect of Si and 24-EBL on the growth, physiology, and metabolic alterations in pea seedlings with and without Cd stresswas investigated by evaluating growth, chl content, photosynthetic efficiency, osmolyte accumulation, antioxidant enzymatic responses, and mineral nutrient contents. Numerous studies have shown that Si and 24-EBL have ameliorative effects against a wide range of abiotic and biotic stresses [2, 34, 38, 39, 65]. However, at low concentration, Si can also increase plant growth and development without any apparent toxicity [66]. In our study, the growth of pea plants declined under Cd stress (Table 1). The subsequent application of Si or 24-EBL, either individually or in combination, augmented the growth attributes in Cd-stressed plants (Table 1). Stimulation of growth by Si in Cd stressed plants has been reported in cucumber [67], wheat [68], cotton [69], and peanut [43]. Similarly, 24-EBL supplementation has improved growth of Cd-stressed plants including tomato [40], radish [65], and bean [41]. Enhanced growth parameters could be due to the ability of 24-EBL to control cell elongation and division via upregulation of xyloglucan endo-transglycosylase [70, 71] or to a dilution effect of Si that decreases metal uptake or increases nutrient uptake by plants, resulting in higher photosynthetic efficiency [8]. Si application has increased both shoot and root DW in many plant species under Cd stress, including maize [72], wheat [6], and rice [73]. Co-application of 24-EBL and Si had pronounced effects on the growth and biomass of pea seedlings under Cd stress likelydue to synergistic or additive effects.

### Silicon and 24-EBL restored pigment content and photosynthetic efficiency

In this study, Cd-stressed pea seedlings had lower chlorophyll and carotenoid contents (Table 2). Reductions in chlorophyll and carotenoid synthesis in response to Cd stress may be due to the inhibitory effect of Cd on the enzymes associated with pigment biosynthesis [74]. Deleterious effects of Cd stress have been reported in maize:Cd reduced chlorophyll synthesis [75], the photochemical quantum yield of photosystem II (Φ_PSII_), and the CO_2_fixation rate [76]. Si application enhanced the chlorophyll pigment and carotenoidcontents of pea seedlings (Table 2). Exogenous application of Si has had positive effects on chlorophyll biosynthesis and photosynthetic machinery in Cd-stressed maize [77], wheat [23], and pea [8]. Wu, et al. [78] reported that Si supplementation reduced Cd translocation in cucumber roots, thereby decreasing the interference of Cd complexation with photosynthetic machinery. Sa [79] found that photosynthetic pigments were restoredfollowing exogenous application of Si in cotton seedlings. Stimulatory effects of Si on photosynthetic processes could be dueto impaired heavy metal uptake, which would facilitate PSI and PSII activation [80]. Si ameliorates the decline in chlorophyll fluorescence by inhibiting Cd uptake as Si induces modifications in Cd binding properties of cell wall [81]. Exogenous application of 500mg SiO_2_to Cd-stressed *Allium sativum* L. seedlings increased the quantum efficiency [82]. Similarly, exogenous application of EBL to Cd-stressed *Raphanus sativus* enhanced growth byimproving the photosynthetic pigment concentration [83]. Foliar application of 24-EBL ameliorated the damage to chlorophyll and carotenoid contents, which supportsthe findings of studies in *Brassica juncea* L. [84] and *Raphanus sativus* L. [85]. Exogenous application of 24-EBL enhanced photosynthetic pigments andcarotenoidcontent due to its stimulatory effect on ribulose 1,5-bisphosphate carboxylase oxygenase activity [86]. EBL has improved photosynthesis by increasing the activity of Calvin cycle enzymes Co-application of EBL+Si to pea seedlings augmented chlorophyll content and carotenoid concentration, probably by modulating mineral uptake, particularly magnesium, which forms an integral part of chlorophyll molecules. Photochemical quenching (qP) and quantum yield of PSII were highest in pea seedlings treated with Si + EBL, suggesting a cumulative stimulatory effect on photosynthetic efficiency with Si improving nutrient uptake and 24-EBL reducing the photo-damage caused by the increase in carotenoid concentration.

### Silicon and 24-EBL modulated physiological status and osmolyte accumulation

Cd-stressed pea seedlings had reduced physiological activities including photosynthetic rate, CO_2_ assimilation rate, stomatal conductance, and transpiration rate (Table 3). Physiological activity decreases with a reduction in enzymatic activity in the Calvin cycle and impaired electron transfer across the electron transport chain [16, 32]. Further, a significant reduction in stomatal conductance and relative water content was noted with Cd stress. Supplementation with Si or 24-EBL enhanced the physiological activities and relative water contentsin the Cd-stressed pea seedlings (Table 3). Si enhanced the activity of gas exchange characteristics, including net photosynthetic rate, stomatal conductance, transpiration rate, and water use efficiency, under Cd stress in cotton, rice, and cucumber [67, 87]. Cadmium stress increased the photosynthetic rate and chlorophyll fluorescence in barley and wheat [88, 89]. Si enhances Cd tolerance by increasing the instantaneous water use efficiency, carboxylation efficiency of ribulose 1,5-bisphosphate carboxylase oxygenase, and light use efficiency [80]. The stimulatory effect of Si on photosynthetic machinery might be due to reduced Cd translocation by plants with less damage to photosynthetic machinery [77]. EBL significantly enhances the photosynthetic rate in Cd-stressed tomato plants by modulating photosynthetic efficiency [90]. Co-application of 24 EBL and Si improvedthe photosynthetic rate, CO_2_ assimilation rate, stomatal conductance, and transpiration rate in Cd-stressed pea seedlings, thereby confirming their stimulatory effects. Relative water content increased with the combined Si and 24-EBL treatment in both control and Cd-stressed pea seedlings. Enhanced relative water content was also observed in Cd-stressed *Phaseolus vulgaris* treated with 24-EBL [41] and was attributed to its inhibitory effect on ABA levels, which might be correlated with the normalization of water relations [91]. Si application increases the relative water content by modulating water use efficiency and stomatal conductance in plants under heavy metal stress [89]. The co-application of Si + EBL modulated physiological processes by up-regulating enzymes associated with metabolic processes in the present study.

Increased proline and GB accumulation is the main plantresponse for maintaining tissue water potential to protect major cell metabolism and functions [92, 93]. In Cd-stressed pea seedlings, proline and GB levels increased significantly when supplemented with Si, EBL,or both. In pea seedlings treated with both Si + EBL, proline accumulated more than GB, which was also observed in drought-stressed sorghum after Si supplementation owing to the activationof the aquaporin gene and transcription factors, thereby facilitating water uptake [94, 95]. Supplementation with 24-EBL increased proline accumulation in aluminum-stressed mung bean seedlings [96], copper-stressed mustard seedlings [86], and cold-stressed peach trees [97]. Gao and coworkers indicated that increased proline levels with EBL supplementation in peach fruit were caused by changes in the P5CS enzyme (D1-pyrroline-5-carboxylate), which activated the proline synthesis pathway, and suppressed proline dehydrogenase activity leading to a reduction in proline consumption. Co-application of Si + EBL enhanced osmolyte accumulation in Cd-stressed plants due to thelikely interactive effect on the upregulation of proline biosynthetic genes [96] and activation of transcription factors related to water relations [94].

### Silicon and 24-EBL reduced hydrogen peroxide MDA contents, and electrolyte leakage and methyl glyoxalase

Our data revealed a significant increase in the generation of H_2_O_2_, MDA, and the rate of electrolyte leakage in Cd-stressed pea seedlings, relative to the control (Table 4). This may be due to Cd-induced free radical generation, which would alter membrane stability, increasing its permeability [16]. Higher ROS generation in response to Cd stress has been reported[1, 16–18, 92]. Enhanced production of H_2_O_2_ might be due to lower RWC, which would impair its distribution from generation sites [98]. In this study, supplementation with EBL and Si, either individually or combined, reduced H_2_O_2_ generation. Si reduced MDA contents, H_2_O_2_ levels, and the electrolytic leakage rates in shoots and roots of Cd-stressed *Pisum sativum* [8], rice [81] and maize [73]. Application of Si reduces free radical generation by maintaining the normalized pool of osmolytes and water content within cells, as observed in Cd-stressed peas [8]. This study confirmed that Si enhances the restoration of damage induced by Cd and improves membrane stability, as reported in Cd-stressed maize [99], cotton [79], cucumber [67], and pea [8]. Application of EBL to Cd-stressed chickpea seedlings reducedthe production of H_2_O_2_, lowered lipid peroxidation, and enhanced membrane stability by lowering the overall ROS generation to protect photosynthetic machinery[90]. One important reason for the reduction in lipid peroxidation and ROS production by EBL might be enhanced endogenous levels of growth hormones such as ethylene and salicylic acid that cross-talk and provide tolerance against metal stress [100]. Co-application of Si+EBL modulated lipid peroxidation, reduced MDA content, and improved membrane stability more effectively than the individual treatments in Cd-stressed pea plants by increasing ROS scavenging activity.

### Silicon and 24-EBL modulated antioxidant activity

Oxidative stress is the main response of plants to varied abiotic and biotic stresses, including heavy metal stress [8, 80, 101–103]. The antioxidant system is the key protagonist in the amelioration of oxidative stress induced by ROS [16, 104]. We evaluated the effect of Si and EBL on the main antioxidants/antioxidant enzymes, including MDHAR, DHAR, AsA, GSH, GSSG, and GST, and enzyme activities (SOD, CAT, APX, and GR) in Cd-stressed seedlings to determine their contribution to oxidative stress management (Table 5). Supplementation with either EBL or Si and the enhancement of Cd-induced antioxidant enzymes was thecrucialstrategy to improve seedling growth under Cdstress. Supplementation with Si enhanced the contents of antioxidants and antioxidant enzymes, in particular, Si significantly modulated CAT activity. Increases in CAT activity can be explained by the inhibition of Cd translocation from the roots to shoots in peas, which is regulated by Fe transport under Si supplementation [8]; this isbecause CAT is a heme-containing antioxidant enzyme that is dependent on the available iron pool of plants [105]. Increases in antioxidants and antioxidant enzymes in response to Cd+Si stress has been observed in numerous plants including pakchoi[106], peanut [107], maize [99], cotton [87], wheat [23], and pea [8]. SOD and GR activities increased in response to Si application, suggesting an improved efficiency in the conversion of O_2_–H_2_O_2_[25]. Si not only facilitates the activation of antioxidant enzymes but also maintains the pool of non-enzymatic antioxidants such as MDHAR, DHAR, AsA, GSH, GSSG, and GST. Wu, et al. [108] also showed that Si-mediated increases in antioxidant enzyme activities might be an adaptive strategy to augment Cd stress in tomato. Increases in the antioxidant pool can be attributed to significant changes in sulfur-containing aminoacids such as cysteine and methionine in response to Si supplementation [8]. Increases in such aminoacids can be directly correlated with the higher pool of GSH in pea seedlings supplemented with Si [8]. Several studies have shown that Si-mediated increases in non-enzymatic antioxidants alleviate Cd stress in various plants such as pakchoi [106] and pea [8]. Supllementation with EBL enhanced antioxidant enzymes as well as non-enzymatic antioxidants in Cd-stressed pea plants (Table 5). Similar findings have been reported where EBL alleviated oxidative stress in *Raphanus sativa* [85], *Brassica juncea* [86], and *Cicer arietinum*[16] under heavy metal stress. EBL-induced enzymatic activities are attributed to the activation of genes implicated in the gene expression of SOD, APX, and CAT activities [31]. Another possible reason for the activation of enzymatic activity might be the BR signaling kinase (BSK 1), which promotes salicylic acid levels that consequently ameliorate the effects of oxidative damage [109]. Increases in GR and GST activities in response to EBL supplementation can be explained by an increment in the GSH pool and significant decline in NADPH oxidase activity, which leads to the alleviation of heavy metal-induced toxicity [84]. In addition to EBL-induced increases in the GSH pool, EBL enhances other antioxidants such as AsA, MDHAR, and DHAR, as reported in pakchoi [110], *Brassica juncea* [86], *Ficus concinna* [111], and *Solanum lycopersicum* [39]. Co-application of EBL+Si might up-regulate biosynthetic genes associated with the activation of enzymatic and non-enzymatic oxidants of the Asc–GSH cycle.

### 24-EBL maintained Gly I and Gly II activities

One of the key strategies of plants under heavy metal stress is to accumulate MG [112–114]. In this study, higher accumulation of MG was an indicator of stress in pea seedlings (Table 6). Higher concentrations of MG lead to the depletion of GSH due to the conversion to hydroxyacylglutathione [115]. Higher levels of MG might be toxic or could result in the depletion of GSH. Supplementation of pea seedlings with Si and EBL individually or in combination resulted in higher accumulation of Gly I and GlyII, which protect plants against Cd stress-induced MG accumulation [116]. Higher MG levels were also noted in mungbean (*Vigna radiata* L.) and rice (*Oryza sativa* L.) in response to Cd and Cu stresses, respectively, relative to the controls[19, 117]. Transgenic plants showed over-expression of GlyI and Gly II, which caused higher influx of MG levels against heavy metal stress via GSH detoxification, consequently reducing lipid peroxidation [118]. In this study, Gly I and Gly II activities increased in response to Cd stress. Similarly, enhanced Gly I activity with Cd and Zn toxicity has been reported in various plant species [112, 115, 119, 120]. A decline in Gly II in response to Cd stress might be due to the proteolytic degradation of enzymes. Supplementation of EBL in *Ficus concinna* maintainedthe pool of Gly I and Gly II against high temperature stress [111]. Application of Si increases Ca uptake, which enhances Gly I and Gly II, and contributes to the decline in Cd-induced growth inhibition [8, 20]. Co-application of Si and EBL increases the uptake of minerals such as Ca and endogenous levels of hormones that are directly implicated in the maintenance of the glyoxalase pool and MG detoxification against Cd stress. Detoxification of MG via glyoxalase is inadequate to combat Cd stress and activate a tolerance strategy. Si and EBL alleviate Cd-induced oxidative stress by maintaining Gly I and Gly II activities, indicating that both facilitate GSH restoration and glutathione redox potential via the glyoxalase system.

### Silicon and 24-EBL reduced Cd accumulation

Due to its higher mobility in soil and plants, Cd is easily absorbed by plant roots (Table 6). Supplementation with Si reduced Cd accumulation in the roots, shoots, and leaves of Cd-stressed pea seedlings (Table 7), which has been reported elsewhere [8, 107]. Si interferes with root uptake and Cd translocation from roots to shoots due to co-precipitation at the root surface, which decreases Cd transport from roots to xylem, and increases Ca uptake, thereby reducing Cd uptake due to competition [8]. The co-precipitation of Cd and Si in cell walls restricts Cd translocation from shoots to grain, which alleviates Cd toxicity and grain contamination. Rahman, et al. [8] revealed that Si supplementation coincides with an increased inflow of S-containing compounds (cysteine, methionine, and glutathione) that contribute to phytochelatin (PC) synthesis in plant tissues. Phytochelatin can function as a second-line defense against Cd stress. Moreover, Si forms silicates within the cytoplasm, leading to the inhibition of symplastic transport of heavy metals [121, 122]. Si application can improve Cd toxicity by increasing plant tolerance to Cd stress. EBL reduced Cd accumulation in roots, shoots, and leaves of pea seedlings (Table 5). Supplementation with EBL reduced Cd accumulation by increasing Ca absorption and maintaining ionic homeostasis [43, 91]. Further, 24-EBL enhances the uptake of K^+^, Ca^2+^, and Mg^2+^ in the roots, and these cations are preferentially transported to younger leaves through vascular tissues to reduce Cd translocation [17]. Moreover, 24-EBL enhances the absorption of essential inorganic ions, reducesthe uptake of toxic ions, and promotes ion homeostasis, especially K^+^/Na^+^, Ca^2+^, and Mg^2+^ in the upper leaves, Ca^2+/^Na^+^and Mg^2+^/Na^+^ in the roots, and K^+^/Na^+^ in the petioles [2, 123]. Hence, the co-application of Si+EBL reduced Cd accumulation by maintaining ion homeostasis, offering better conditions for osmotic adjustment, and blocked Cd uptake by co-precipitation.

### Silicon and 24-EBL enhanced mineral uptake

Mineral nutrition is crucial for plant growth and development. Our study showed that cadmium impairs mineral uptake in pea seedlings (macro, Table 8; micro, Table 9), more so in shoots than roots. Cadmium impairs mineral absorption in other species including beans [41, 124], tomato [125], and *Arabidopsis thaliana* [126]. In this study, Si supplementation significantly increased macro- and micronutrient levels in the shoots and roots of control and Cd-stressed pea seedlings. Si supplementation maintains phosphorus homeostasis by regulating the uptake and overload of phosphorus from soil [127], and improves potassium uptake by activating H-ATPase even at low concentrations [127, 128]. Abdel Latef and Tran [129] have shown increased nitrogen and calcium uptake in crops after supplementation with sodium metasilicate. Si increases the oxidizing power of roots, thereby preventing surplus uptake of iron and limiting iron toxicity [130]. Further, Si regulates iron uptake from acidic soils via the release of OH through plant roots when plantsare supplemented with Si [110]. Supplementation with Si influences the solubility of various elements such as P, K, and Ca and hinders the uptake of toxic metals such as Cd, As, and Cu in rice grains [131]. Si supplementation alters phosphorus precipitation along with Fe and Mn in potato plants [132]. Tripathi, et al. [133] found that Si enhanced macro- (Mg, Ca, and K) and micronutrients (Zn and Fe) in rice seedlings under chromium stress. Supplementation with EBL increased both macro- and micronutrient uptake in Cd-stressed and control pea plants. In cucumber seedlings, EBL application improves nitrogen metabolism by maintaining ion homeostasis through the excessive flow of Ca^2+^ and Mg^2+^ into shoots and roots [134]. EBL increases Fe uptake by enhancing ferric chelate reductase activity, thereby enhancing Fe (III) reduction to Fe (II) and consequently increasing Fe content in cucumber seedlings [135]. Foliar application of EBL increased H^+^-ATPase activity resulting in a surplus of Fe in plants. Application of EBL activates both H^+^-ATPase and Ca^2+^-ATPase in roots and leaves of Fe-deficient pea plants [136]. H^+^-ATPase can establish an electrochemical potential gradient to maintain ion balance in plants [137], and Ca^2+^functions as an intracellular messenger in coupling an extensive range of extracellular signals to explicit responses [138], thus enhancing ion uptake and translocation. Co-application of EBL+ Si augmented mineral nutrition by decreasing the uptake of toxic metals and increasing cation exchange capacity.

## Conclusion

Cd stress induces numerous physiological and biochemical processes that inhibit plant growth and metabolism. These toxic effects were ameliorated in Cd-stressed pea seedlings by supplementation with Si or EBL, or a combination of the two, which was more effective. The potential mechanism for the favorable effects of Si and EBR are summarized as follows: (1) restoration of chlorophyll and physiological activities such as photosynthetic efficiency, stomatal conductance, CO_2_ assimilation rate, stomatal conductance, and transpiration rate;(2) decline in Cd root-to-shoot translocation; (3) increasein antioxidant enzyme activity and generation of antioxidant molecules such as AsA and GSH; (4) higher accumulation of osmolytes such as proline and GB; (5) improved mineral uptake resulting in higher physiological activity; and (6) detoxification of MG via the glyoxalase system. The results of our study indicate that co-application of Si+ EBL is an eco-friendly way for improving the performance of plants under Cd stress. Further studies are needed to elucidate the mechanism underlying the interactive effect of Si +EBL in Cd detoxification. These findings might provide further potential for the relevance of Si and EBL in phytoremediation and Cd detoxification in crops.
